# Comparative efficacy and safety of pegylated interferon-alpha monotherapy vs combination therapies with entecavir or tenofovir in chronic hepatitis B patients

**DOI:** 10.1128/spectrum.02694-24

**Published:** 2025-04-02

**Authors:** Huiqing Liang, Xiaoting Zheng, Qianguo Mao, Jiaen Yang, Qingfa Ruan, Chuncheng Wu, Yaoyu Liu, Siyan Chen, Luyun Zhang, Manying Zhang, Hongli Zhuang, Li Lin, Shaodong Chen

**Affiliations:** 1 Hepatology Unit, Xiamen Hospital of Traditional Chinese Medicine, Xiamen, Fujian, China; 2 School of Clinical Medicine, Fujian University of Traditional Chinese Medicine, Fujian, China; 3 Department of Traditional Chinese Medicine, First Affiliated Hospital of Xiamen University, Xiamen, Fujian Province, China; 4 School of Medicine, Xiamen University, Xiamen, Fujian Province, China; Penn State College of Medicine, Hershey, Pennsylvania, USA

**Keywords:** antiviral drugs, chronic hepatitis B, combination therapy, entecavir, tenofovir

## Abstract

Current treatments for chronic hepatitis B (CHB) virus involve nucleos(t)ide analogs and pegylated interferon-alpha (PEG-IFNα). This study compares the efficacy and safety of PEG-IFNα monotherapy with its combinations with entecavir (ETV) and tenofovir disoproxil fumarate (TDF) in managing CHB. We included 147 treatment-naïve patients divided into three groups: Group A (PEG-IFNα-2b with ETV), Group B (PEG-IFNα-2b with TDF), and Group C (PEG-IFNα-2b monotherapy). Evaluations occurred every 12 weeks up to 48 weeks. The Kaplan-Meier method showed no significant differences in cumulative HBsAg loss, but HBV DNA clearance rates were higher in the TDF group than in the ETV group (*P* = 0.01). Higher incidences of elevated alanine aminotransferase (ALT), aspartate aminotransferase, and thrombocytopenia were observed in the TDF group compared to other groups. After propensity score matching, the TDF group had a higher undetectable HBV DNA rate than the IFN group, but no significant differences in HBsAg clearance rates. Both TDF and ETV groups achieved more significant HBsAg reductions from baseline to week 48 than the IFN group (*P* < 0.05). ETV showed a lower HBeAg clearance rate (30.00% vs 87.50%, *P* < 0.05) but higher ALT normalization (76.92% vs 45.45%, *P* < 0.05). In the TDF group, patients with lower baseline HBsAg levels, high ALT levels, and lower aspartate aminotransferase-to-platelet ratio index (APRI) scores were more likely to achieve HBsAg loss. These findings suggest that TDF and ETV are effective for viral suppression, with TDF showing superior HBV DNA clearance but more adverse events.

**IMPORTANCE:**

This study investigates how different treatments for chronic hepatitis B (CHB), a widespread liver infection, compare in effectiveness and safety. By evaluating the use of pegylated interferon-alpha alone and in combination with two other drugs, entecavir and tenofovir disoproxil fumarate (TDF), researchers found that TDF offers better viral suppression but also comes with more side effects. For patients receiving TDF combined with PEG-IFN therapy, low HBsAg levels, elevated alanine aminotransferase levels, and lower APRI scores were associated with a higher likelihood of achieving HBsAg loss. Consistent with previous findings, this study confirms the benefits of nucleos(t)ide analog plus PEG-IFN therapy for CHB treatment and further explores which patients are more likely to benefit from combination therapy. Furthermore, this study underscores the importance of further monitoring adverse events in patients receiving combination therapy.

## INTRODUCTION

Hepatitis B virus (HBV) infection remains a major global health concern (1). In 2019, the global prevalence of chronic HBV (CHB) infection was 4.1%, representing 316 million people living with HBV (2, 3). CHB infection can also lead to severe liver diseases such as cirrhosis, liver failure, and hepatocellular carcinoma (HCC) (4). Despite the global introduction of HBV vaccination programs, CHB continues to pose a significant public health burden, particularly among individuals already infected (5). Consequently, there is an increasing focus on developing and refining antiviral therapies to manage the infection and reduce its complications, including liver cancer (6).

Current treatment strategies for CHB primarily involve two therapeutic approaches: nucleos(t)ide analogs (NAs) and pegylated interferon-alpha (PEG-IFNα) (7). NAs, such as entecavir (ETV), tenofovir disoproxil fumarate (TDF), and the newer tenofovir alafenamide (TAF), are widely used due to their ability to suppress HBV replication effectively (8). These antiviral agents function by inhibiting the reverse transcription of HBV DNA, thereby reducing viral load and slowing disease progression (9). PEG-IFNα, on the other hand, not only has antiviral properties but also modulates the immune response, potentially aiding in the clearance of HBV-infected cells (10). However, the use of PEG-IFNα alone is limited by its lower response rates and significant side effects, negatively impacting patient compliance and tolerability. Studies show that only around 30% of patients achieve a sustained virological response with interferon treatment, and its use is often confined to patients who prefer a finite treatment duration or those who are not suitable for long-term NA therapy (11, 12).

In addition to monotherapy, combination therapy with PEG-IFNα and NAs has been widely applied as a practical approach for managing CHB, particularly in patients with poor responses to initial treatment (13). PEG-IFNα, when combined with NAs such as ETV or TDF, may offer additional synergistic benefits, improving viral suppression and enhancing immune control (14–16). However, several unresolved issues still exist regarding the use of PEG-IFNα in combination with NAs for the treatment of CHB. One of these issues is identifying which patients stand to benefit most from combination therapy, which may be essential not only for optimizing treatment efficacy but also for minimizing potential side effects and developing personalized treatment strategies (16).

Regarding safety, ETV has been reported to have a low resistance rate and is effective in treatment-naive patients, while TDF has demonstrated durable viral suppression with no documented resistance after five years of continuous therapy (17–19). Despite the efficacy of these therapies, some concerns remain, such as potential bone and renal toxicity with long-term TDF use (20, 21). These safety concerns underscore the importance of thoroughly evaluating therapeutic strategies in different populations to ensure long-term safety.

Given the limitations of monotherapy with either PEG-IFNα or NAs, and the potential benefits of combination therapy, there is a growing need for studies that directly compare the efficacy of these treatment strategies. The aim of the present study is to evaluate the effectiveness and safety of three different therapeutic approaches in patients with HBV infection based on real-world data: PEG-IFNα monotherapy, PEG-IFNα combined with ETV, and PEG-IFNα combined with TDF. In addition, the study will focus on exploring which patients would benefit more from combination therapy.

## MATERIALS AND METHODS

### Study population

In this study, a total of 204 antiviral therapy-naive CHB patients were enrolled from the Center of Liver Diseases at Xiamen Traditional Chinese Medicine Hospital from December 2020 to June 2023. These patients were repeatedly examined every 12 weeks.

The inclusion criteria were as follows: (i) age between 18 and 70 years, (ii) HBsAg positive for at least 6 months, (iii) serum HBV DNA positive, and (iv) voluntarily signed an informed consent form to participate in the study. The exclusion criteria were as follows: (i) patients with acute hepatitis or severe chronic hepatitis, (ii) co-infection with other hepatitis viruses, such as HAV, HCV, HDV, or HEV, (iii) co-existing autoimmune hepatitis, primary HCC, or other chronic liver diseases, and (iv) pregnant women. The dropout criteria applied were as follows: (i) poor treatment compliance, defined as less than 48 weeks of therapy, (ii) occurrence of severe complications or adverse reactions during the surveys, and (iii) voluntary withdrawal from the study.

### Therapeutic protocols

This study is a real-world prospective study. Patients were assigned into three treatment groups based on their baseline HBV infection status in this study: Group A received a combination of PEG-IFNα-2b (Pegbin, Xiamen Amoytop Biotech Co., Ltd.) and entecavir (RuiFuEN, Suzhou Dawnrays Pharmaceutical Co., Ltd.), Group B received PEG-IFNα-2b and tenofovir (BeiXin, Brilliant Pharmaceutical Co., Ltd.), and Group C (control group) received PEG-IFNα-2b alone. In all groups, PEG-IFNα-2b was administered subcutaneously at a dose of 180 µg per injection once weekly for at least 48 weeks. Entecavir was taken orally at a dose of 0.5 mg once a day, and tenofovir was taken orally at a dose of 300 mg once a day for at least 48 weeks.

During the 48 weeks treatment period, all participants underwent clinical and safety assessments every 12 weeks. Baseline information, including sex, age, family history of hepatitis B or liver cancer, fatty liver status, and alcohol consumption history, was collected via a questionnaire. At each visit, the following tests were performed to evaluate treatment safety and potential adverse events: electrocardiogram, liver and kidney function tests, complete blood count, and urinalysis. Serum HBV DNA, HBsAg, alanine aminotransferase (ALT), aspartate aminotransferase (AST), total bilirubin, and alpha-fetoprotein (AFP) were measured at every visit.

### Laboratory measurements

In this study, serum HBV DNA levels were quantified using a fluorescence quantitative PCR. Serum hepatitis B markers, including HBsAg, anti-HBs (HBsAb), HBeAg, anti-HBe (HBeAb), and anti-HBc (HBcAb), were measured using an electrochemiluminescence immunoassay. Biochemical tests, including ALT and AST, were conducted on the automatic biochemical analyzer. Serum AFP levels were determined by enzyme-linked immunosorbent assay.

Based on these measurements, the FIB-4 index and APRI index were calculated to assess liver fibrosis, with the following formulas: FIB-4 = age (years) × AST (U/L)/(platelets [10^9^/L] × ALT [U/L]^½^) and APRI = (AST /ULN) / (PLT [10^9^/L]) × 100, where ULN represents the upper limit of normal for AST (40 IU/L) (22, 23).

### Outcomes

The primary efficacy endpoints were HBsAg loss (<0.05 IU/mL) and undetectable serum HBV DNA (<20 IU/mL) as measured by fluorescence quantitative PCR. Clinical cure was defined as HBsAg clearance, with or without seroconversion, undetectable HBV DNA, and normalization of ALT and AST levels. HBsAg seroconversion was defined as HBsAg < 0.05 IU/mL and HBsAb > 10 mIU/mL. The biochemical response was defined as the normalization of ALT (<50 U/L) or AST (<40 U/L). Biochemical response was analyzed only in patients with baseline ALT > 50 U/L or AST > 40 U/L.

### Statistical analysis

HBV DNA and HBsAg concentrations were log10 transformed before statistical evaluation. Patient characteristics were presented as mean ± standard deviation (SD) for continuous variables. For the comparative analysis between two therapy groups, independent Student’s *t*-tests were used for continuous variables, while the *χ*^2^ test was applied for categorical variables. To compare continuous variables across three groups, a one-way analysis of variance (ANOVA) was employed, whereas categorical variables were analyzed using the *χ*^2^ test for larger sample sizes or Fisher’s exact test when sample sizes were smaller or when expected cell frequencies were less than 5.

Next, in the entire analysis population, based on the longitudinal data (collected in 0, 12, 24, 36, and 48 weeks), the Kaplan-Meier method was applied to estimate the cumulative probability of the primary efficacy endpoints, and inter-group comparisons were performed using the log-rank test to determine statistical significance between survival curves.

Propensity score matching was then applied to reduce selection bias due to differences in baseline characteristics among the three treatment groups. Logistic regression was used to generate propensity scores based on key covariates such as age, gender, baseline HBeAg status, serum HBV DNA levels, and serum HBsAg levels. A caliper width of 0.3 was set for the matching procedure, ensuring that closely matched pairs were selected. Similarly, Kaplan-Meier survival analysis was conducted in the matched three groups for the primary efficacy endpoints, and intergroup comparisons were performed using the log-rank test. Additionally, mean changes at weeks 24 and 48 for these markers were analyzed using an ANOVA test, followed by *post hoc* comparisons where appropriate.

To identify predictors of HBsAg loss, univariate Cox proportional hazards models were initially performed, followed by multivariate Cox regression to control for potential confounders. Covariates included in the multivariate analysis were chosen based on their clinical relevance and statistical significance in the univariate analysis.

All statistical tests were two-sided, and *P* values less than 0.05 were considered statistically significant. The statistical analyses were conducted using R software (version 4.1.2, http://cran.r-project.org/).

## RESULTS

### Characteristics of the study population

**Fig 1:**
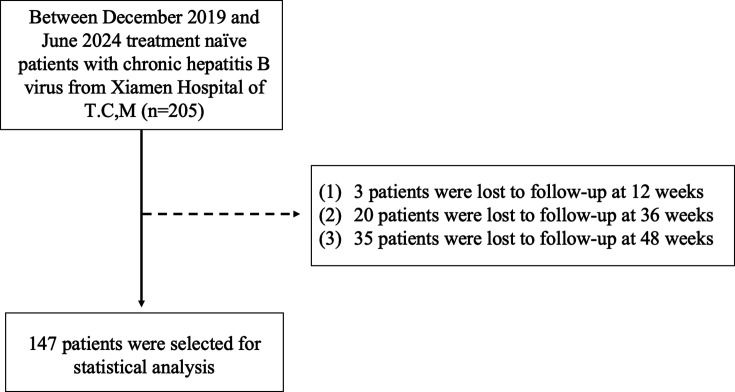
Flow diagram of patients in this study.

After applying the inclusion and exclusion criteria, 147 patients were included in the study. Group A (PEG-IFNα-2b + ETV) consisted of 31 patients (21.1%), Group B (PEG-IFNα-2b + TDF) included 59 patients (40.1%), and Group C (PEG-IFNα-2b monotherapy) had 57 patients (38.8%) (Fig. 1).

The baseline characteristics of the patients are presented in Table 1. The baseline HBV DNA levels were 6.19 ± 2.16 log10 IU/mL in Group A and 5.74 ± 2.29 log10 IU/mL in Group B, both of which were significantly higher than the baseline HBV DNA level of 4.90 ± 2.15 log10 IU/mL observed in Group C (*P* = 0.010 for Group A and 0.043 for Group B). No statistically significant difference was observed between Group A and Group B for HBV DNA levels at baseline. For baseline HBsAg levels, the mean levels were 3.89 ± 0.77 log10 IU/mL in Group A, 3.51 ± 1.25 log10 IU/mL in Group B, and 3.06 ± 1.19 log10 IU/mL in Group C. Similarly, both Group A and Group B exhibited significantly higher baseline HBsAg levels compared to Group C (*P* < 0.001 for Group A and 0.046 for Group B), while no significant difference was found between Group A and Group B.

**TABLE 1:** Baseline characteristics*^a^*^,^*^b^*

	Group A	Group B	Group C	*P*
*N*	31	59	57	
Age, mean (SD), years	37.39 ± 9.00	38.37 ± 8.18	38.44 ± 8.84	0.842
Sex				
Female	10 (32.3)	19 (32.2)	10 (17.5)	0.145
Male	21 (67.7)	40 (67.8)	47 (82.5)	
BMI, mean (SD), kg/m^2^	23.89 ± 4.43	23.71 ± 3.28	23.45 ± 3.36	0.846
Family history of HBV	9 (29.0)	14 (24.6)	18 (30.5)	0.765
Family history of cancer	9 (29.0)	10 (17.5)	15 (25.4)	0.410
Fatty liver	14 (45.2)	25 (42.4)	18 (31.6)	0.350
HBV DNA, mean (SD), log10 IU/mL	6.19 ± 2.16	5.74 ± 2.29	4.90 ± 2.15	0.022
HBsAg, mean (SD), log10 IU/mL	3.89 ± 0.77	3.51 ± 1.25	3.06 ± 1.19	0.004
HBeAg status				
Negative	11 (35.5)	30 (50.8)	43 (75.4)	0.001
Positive	20 (64.5)	29 (49.2)	14 (24.6)	
ALT, mean (SD), U/L	108.65 ± 128.31	100.85 ± 93.53	117.82 ± 166.87	0.791
AST, mean (SD), U/L	62.55 ± 59.49	56.22 ± 49.06	65.68 ± 89.65	0.759
PLT, mean (SD), 10^9^/L	206.17 ± 60.27	210.00 ± 56.29	219.84 ± 57.38	0.505
FIB-4, mean (SD)	1.21 ± 0.69	1.15 ± 0.77	1.06 ± 0.70	0.638
APRI, mean (SD)	0.86 ± 0.89	0.71 ± 0.65	0.79 ± 1.06	0.738

^*a*^ Variables are expressed as mean (standard deviation) or *n* (%).

^*b*^ Group A: PEG-IFNα-2b + ETV, Group B: PEG-IFNα-2b + TDF, Group C: PEG-IFNα-2b.

### HBsAg and HBV DNA clearance

**Fig 2:**
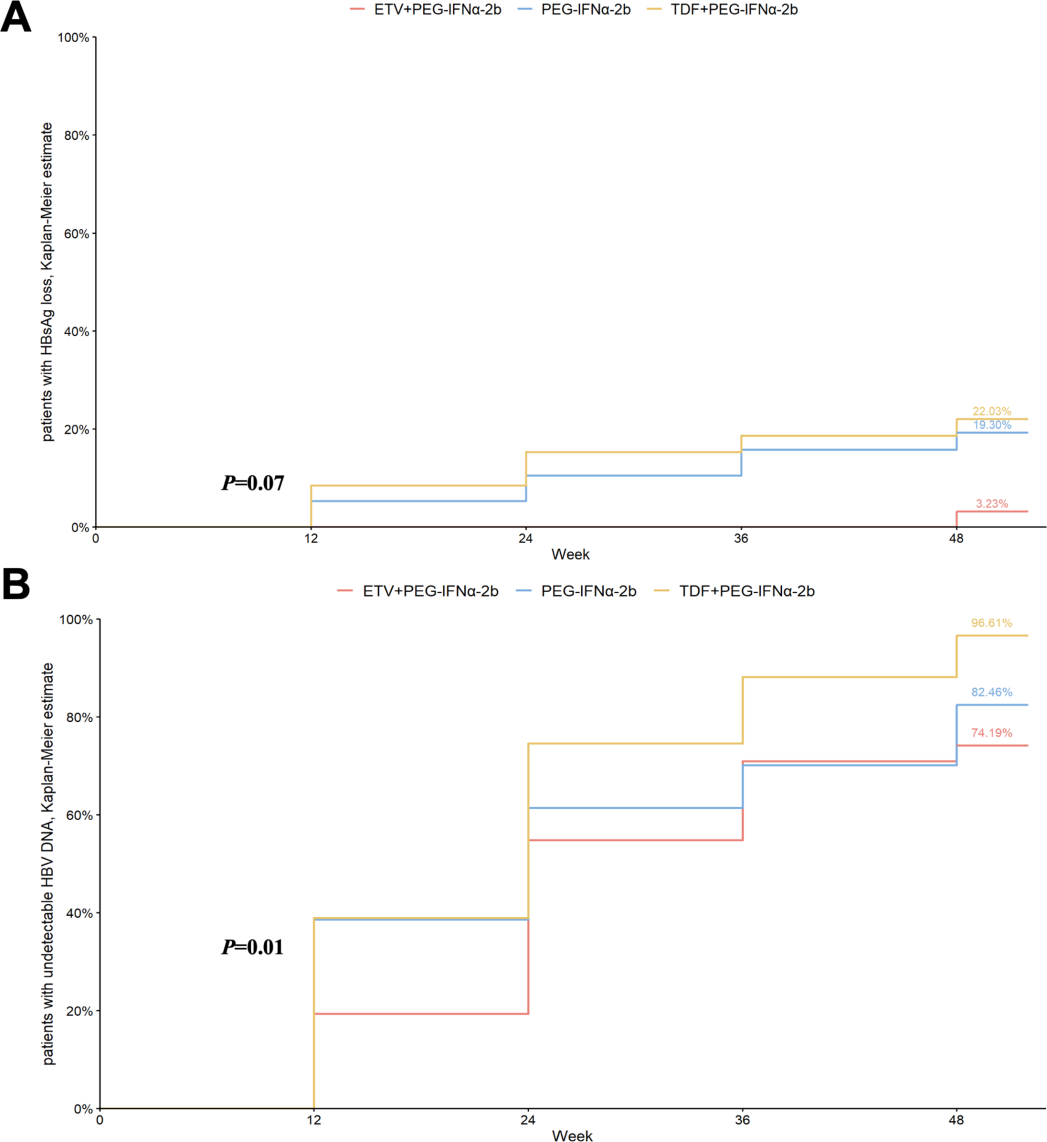
Cumulative probability of HBsAg (A) and HBV DNA (B) loss according to antiviral therapy in the entire study population.

The cumulative probability of HBsAg loss among the three treatment groups is shown in Fig. 2. During the study period, 13 patients (22.03%) in the TDF combination group, 1 patient (3.22%) in the ETV combination group, and 10 patients (17.54%) in the PEG-IFN monotherapy group achieved HBsAg clearance. At week 48 of treatment, the cumulative probability of HBsAg clearance was 22.03% in the TDF combination group, compared to 3.23% and 19.30% in the ETV combination group and the monotherapy group, respectively. The difference among the three groups was not statistically significant (*P* = 0.07, log-rank test).

During the study, only one patient in the PEG-IFN monotherapy group who initially achieved HBsAg (week 24) clearance experienced HBsAg relapse (week 48).

As for HBV DNA clearance, throughout the study, 49 patients (83.05%) in the TDF combination group, 21 patients (67.74%) in the ETV combination group, and 41 patients (71.92%) in the PEG-IFN monotherapy group achieved HBV DNA clearance. At week 48, the cumulative probability of HBV DNA clearance was 96.61% in the TDF combination group, 74.19% in the ETV combination group, and 82.46% in the monotherapy group. The difference was significant (*P* = 0.01, log-rank test) among the three groups. Pairwise comparisons showed a significant difference between the TDF combination group and the ETV combination group (*P* = 0.01, log-rank test), while not for the differences between the monotherapy group and the other two combination groups (both *P* > 0.05, log-rank test) (Fig. 2).

**Fig 3:**
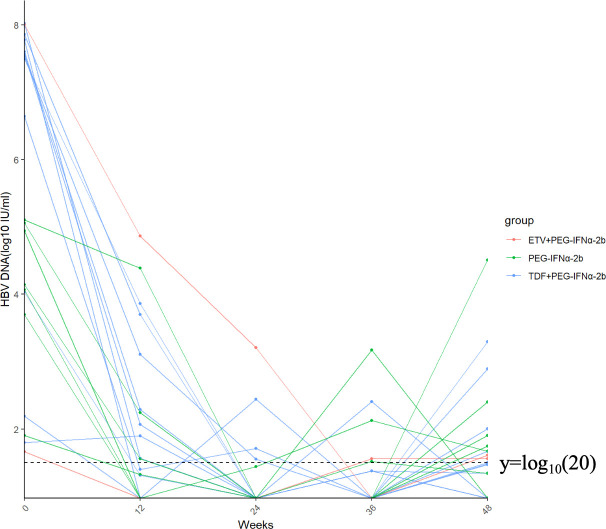
Cumulative probability of patients with undetectable HBV DNA according to antiviral therapy in the entire study population.

During the study period, 20 patients who had achieved HBV DNA clearance experienced HBV DNA relapse, including 2 patients in the ETV combination group, 11 in the TDF combination group, and 7 in the PEG-IFN monotherapy group. Of these, four patients (three in the TDF combination group and one in the monotherapy group) finally achieved HBV DNA clearance by the end of the 48 weeks treatment period (Fig. 3).

### Trimatch and the outcome according to antiviral agents

**Fig 4:**
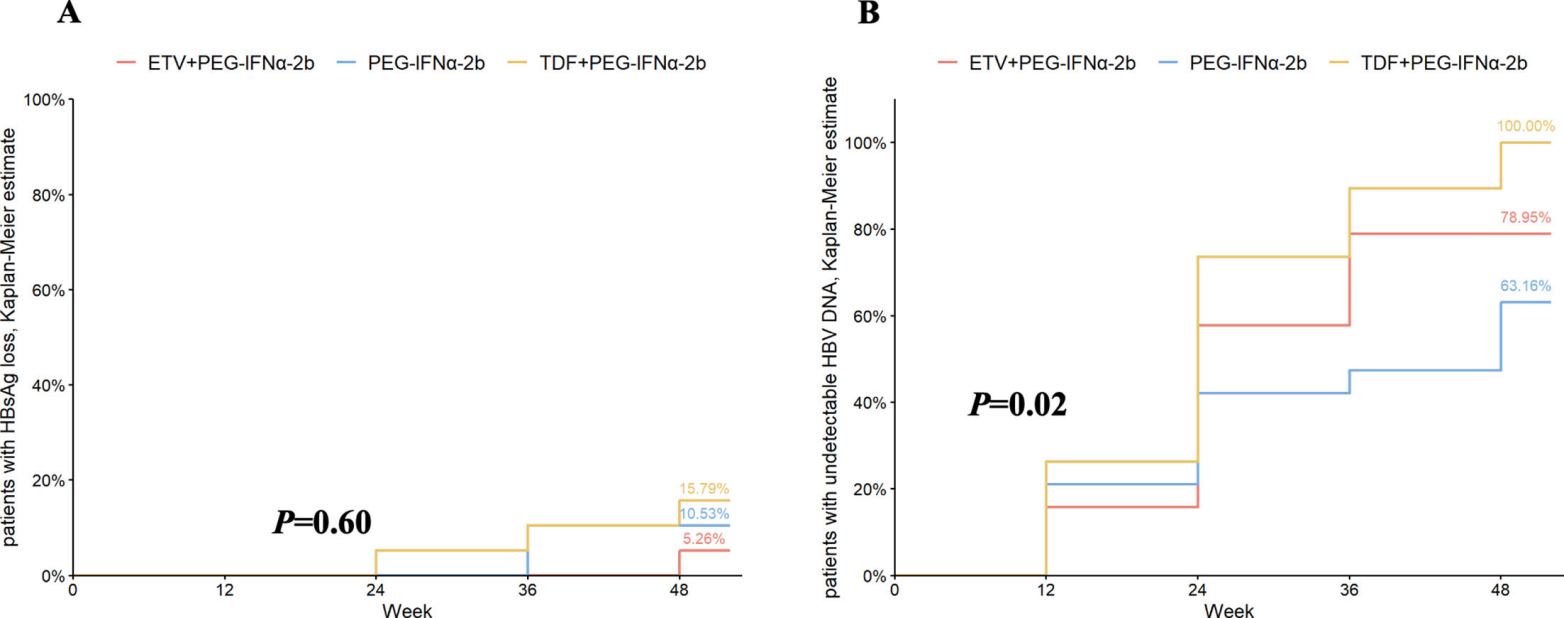
Cumulative probability of (**A**) patients with HBsAg loss and (**B**) with undetectable HBV DNA according to antiviral therapy after trimatch.

After propensity score matching, baseline characteristics were comparable across the three groups (Table S1). After matching, the cumulative probability of HBV DNA clearance was 100% in the TDF combination therapy group at week 48, compared to 78.95% in the ETV combination therapy group and 63.16% in the IFN monotherapy group. Consistent with the analysis in the entire analysis population, the cumulative probability of HBV DNA clearance differed significantly only between the TDF combination group and the IFN monotherapy group (*P* = 0.01, log-rank test), and the cumulative probability of HBsAg loss did not differ significantly among the three groups (*P* = 0.60, log-rank test) (Fig. 4).

**Fig 5:**
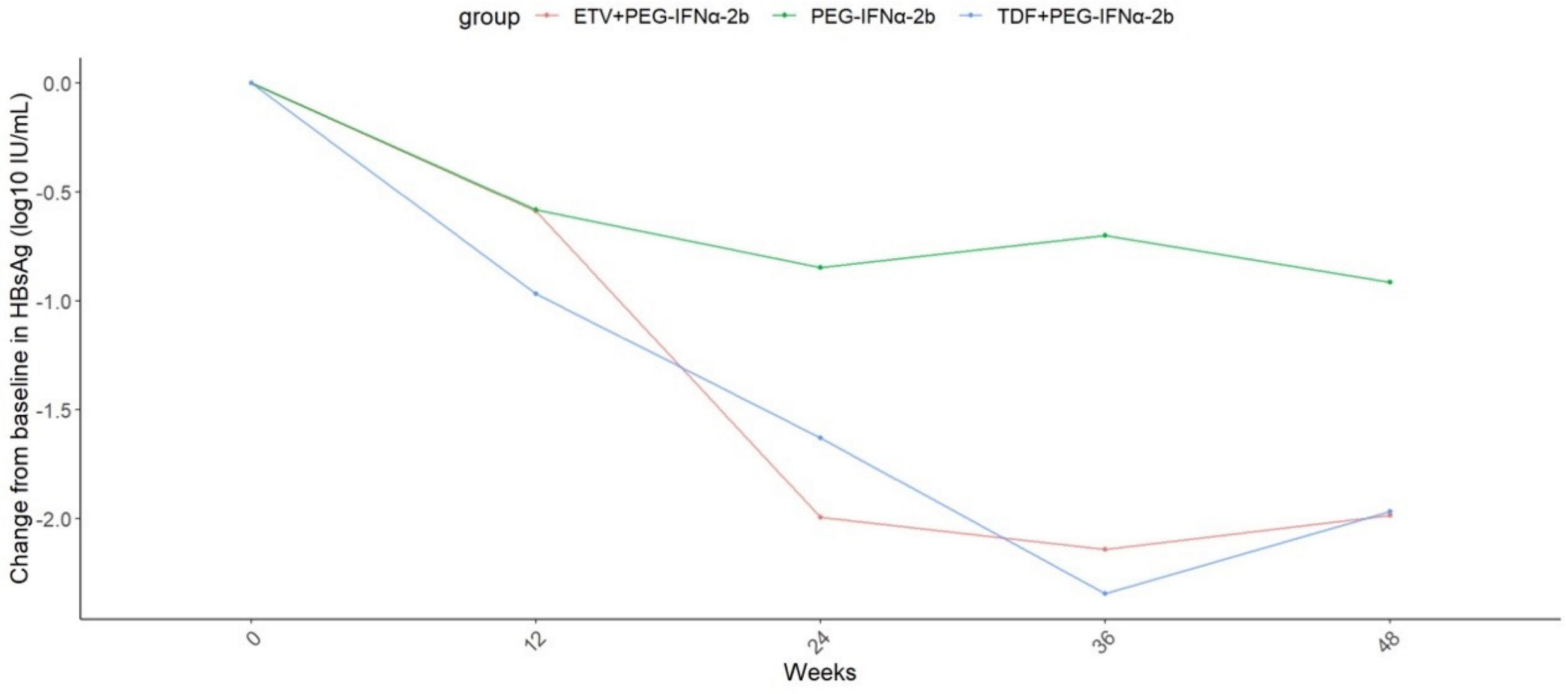
Change in HBsAg level (log10 IU/mL) from baseline to week 48 stratified by different therapeutic protocols after trimatch.

Figure 5 illustrates the changes in HBsAg levels from baseline during treatment across the three groups after matching. Starting from week 24, the mean reduction in HBsAg levels in both the ETV and TDF combination therapy groups was significantly higher than in the IFN monotherapy group (*P* < 0.05 for both), with no significant difference between the ETV and TDF combination groups. By week 48, the mean HBsAg reduction was 1.99 log10 IU/mL in the ETV combination group, 1.97 log10 IU/mL in the TDF combination group, and 0.91 log10 IU/mL in the IFN group.

Efficacy analysis revealed that the HBeAg clearance rate in the ETV combination group was lower than in the IFN group (30% vs 87.50%, *P* < 0.05). In comparison, the ALT normalization rate was higher in the ETV combination group compared to the IFN group (76.92% vs 45.45%, *P* < 0.05). The TDF combination group did not exhibit statistically significant differences when compared to the other two groups (Table 2). Efficacy results for all participants in weeks 24 and 48 are presented in Table S2.

**TABLE 2:** Efficacy results at weeks 24 and 48 after trimatch^*a*^

Response	ETV + PEG-IFNα-2b (*n* = 19)	TDF + PEG-IFNα-2b (*n* = 19)	PEG-IFNα-2b (*n* = 19)
HBsAg loss, *n*/*N* (*%*)			
Week 24	0/19	1/19 (5.26)	0/19
Week 48	1/19 (5.26)	3/19 (15.79)	2/19 (10.53)
*P* value at week 48			
vs PEG-IFNα-2b	1	1	
vs TDF + PEG-IFNα-2b	0.604		
HBsAg seroconversion, *n*/*N* (%)			
Week 24	0/19	0/19	0/19
Week 48	0/19	3/19 (15.79)	2/19 (10.53)
*P* value at week 48			
vs PEG-IFNα-2b	0.486	1	
vs TDF + PEG-IFNα-2b	0.230		
HBV DNA < 20 IU/mL, *n*/*N* (%)			
Week 24	11/19 (57.89)	14/19 (73.68)	7/19 (36.84)
Week 48	14/19 (73.68)	16/19 (84.21)	11/19 (57.89)
*P* value at week 48			
vs PEG-IFNα-2b	0.495	0.151	
vs TDF + PEG-IFNα-2b	0.693		
Functional care, *n*/*N* (*%*)			
Week 24	0/19	1/19 (5.26)	0/19
Week 48	1/19 (5.26)	3/19 (15.79)	2/19 (10.53)
*P* value at week 48			
vs PEG-IFNα-2b	1	1	
vs TDF + PEG-IFNα-2b	0.604		
HBeAg loss, *n*/*N* (*%*)			
Week 24	4/10 (40.00)	6/8 (75.00)	8/8 (100.00)
Week 48	3/10 (30.00)	5/8 (62.50)	7/8 (87.50)
*P* value at week 48			
vs PEG-IFNα-2b	**0.025**	0.569	
vs TDF + PEG-IFNα-2b	0.342		
ALT normalization, *n*/*N* (*%*)			
Week 24	7/13 (53.85)	5/11 (45.45)	4/13 (45.45)
Week 48	10/13 (76.92)	4/11 (36.36)	4/13 (45.45)
*P* value at week 48			
vs PEG-IFNα-2b	**0.047**	1	
vs TDF + PEG-IFNα-2b	0.095		
AST normalization, *n*/*N* (*%*)			
Week 24	4/11 (36.36)	5/10 (50.00)	2/9 (22.22)
Week 48	8/11 (72.73)	3/10 (30.00)	4/8 (50.00)
*P* value at week 48			
vs PEG-IFNα-2b	0.376	0.631	
vs TDF + PEG-IFNα-2b	0.086		

^*a*^ *P* < 0.05 are shown in bold.

### Adverse events

Table 3 summarizes the incidence of adverse reactions across the three treatment groups (ETV combination, TDF combination, and IFN-α monotherapy) and their respective outcomes at 48 weeks.

**TABLE 3:** Adverse events

Variables	ETV combination		TDF combination		IFN-α	
	At least one occurrence	At week 48	At least one occurrence	At week 48	At least one occurrence	At week 48
ALT elevation	9/14	3/14	19/21	7/21	16/22	8/22
AST elevation	13/16	7/16	21/25	13/25	27/33	12/33
Total bilirubin elevation	0/27	0/27	2/54	0/54	2/52	1/52
Direct bilirubin elevation	2/7	1/7	8/23	3/23	7/18	1/18
Thrombocytopenia	13/30	4/30	24/58	10/58	20/56	10/56
Dyslipidemia	1/4	0/4	6/7	2/7	3/8	2/8

ALT and AST elevations were frequent in all groups. In the ETV combination group, 9/14 and 13/16 patients experienced ALT and AST elevations, with three and seven cases persisting at 48 weeks, respectively. The TDF combination group showed a higher incidence, with 19/21 and 21/25 patients affected by ALT and AST elevations, with 7 and 13 cases ongoing at 48 weeks. Similarly, the IFN-α group saw 16/22 and 27/33 patients affected by ALT and AST elevations, with 8 and 12 cases persisting at the end of the study.

No cases of total bilirubin elevation occurred in the ETV combination group. In contrast, the TDF and IFN-α groups had 2/54 and 2/52 cases, respectively, with only one case persisting in the IFN-α group by week 48. For direct bilirubin elevation, 2/7 patients in the ETV combination group were affected, with one case remaining at 48 weeks, while the TDF (8/23) and IFN-α (7/18) groups showed higher incidences, with three and one case(s) still present at the end of the study. Thrombocytopenia occurred in 13/30 patients in the ETV combination group (four persisting at 48 weeks), 24/58 in the TDF combination group (10 persisting), and 20/56 in the IFN-α group (10 persisting). Lastly, dyslipidemia was reported in 1/4 patients in the ETV combination group (none persisting), 6/7 in the TDF combination group (two persisting), and 3/8 in the IFN-α group (two persisting).

Above all, adverse events were observed across all treatment groups, with varying incidence and persistence rates. Notably, the TDF combination group exhibited higher rates of certain adverse events, such as ALT and AST elevations and thrombocytopenia, than the ETV combination and IFN-α monotherapy groups.

### Predictors of HBsAg loss

As above, TDF plus PEG-IFNα therapy showed better outcomes, and we performed univariate and multivariate Cox regression analyses to identify independent predictors of HBsAg loss and to select patients who would benefit more from the combination (Table 4). In the univariate Cox regression, factors including age, baseline HBV DNA levels, baseline HBsAg levels, baseline AST levels, baseline ALT levels, and baseline APRI levels were all independently associated with the risk of HBsAg loss (all *P* < 0.05). The multivariate Cox regression analysis indicated that family history of cancer, baseline levels of HBsAg, ALT, and APRI were still significantly associated with HBsAg loss (Table 4). Patients with lower baseline HBsAg levels, high ALT levels, and lower APRI scores were more likely to achieve HBsAg loss.

**TABLE 4:** Cox regression analysis of factors associated with hepatitis B surface antigen loss in the TDF combination therapy group

Variables	Univariate	Multivariate		
	*P*	HR^*a*^	95% CI	*P*
Sex	0.757			
Age	**<0.001**	0.95	0.90–1.01	0.109
Family history of HBV	0.927			
Family history of cancer	**0.004**	0.02	0.00–0.10	0.203
Fatty liver	0.537			
Drink	0.418			
Baseline HBV DNA, log10 IU/mL	**<0.001**	1.23	0.75–2.01	0.415
Baseline HBsAg, log10 IU/mL	**<0.001**	0.17	0.02–0.25	**<0.001**
Baseline HBeAg status	0.995			
Baseline AST, U/L	**<0.001**	1.02	0.94–1.10	0.722
Baseline ALT, U/L	**<0.001**	1.05	1.02–1.07	**<0.001**
Baseline FIB-4	0.632			
Baseline APRI	**<0.001**	0.01	0.00–0.02	**<0.001**

^*a*^ HR: hazard ratio.

^*b*^ Significance results ( *P*<0.05) are bolded.

## DISCUSSION

In this study, we investigated the efficacy and safety profiles of IFN monotherapy, ETV combining IFN therapy, and TDF combining IFN therapy in patients with CHB. Our findings revealed several important aspects of treatment efficacy, particularly regarding HBsAg clearance, HBV DNA suppression, and biochemical responses.

### Virological efficacy

Based on our findings, although no statistically significant difference in the cumulative probability of HBsAg clearance was observed among the three treatment groups, a significant difference in HBV DNA clearance was noted between the TDF combination therapy group and the ETV combination therapy group. This finding indicates that while HBsAg clearance may be less responsive to differences in treatment regimens, HBV DNA clearance is more dependent on the antiviral efficacy of TDF compared to ETV. Although PEG-IFNα-2b and NAs, such as TDF and ETV, are currently the recommended first-line agents for suppressing HBV replication in patients with chronic hepatitis B, the efficacy of these treatments in achieving HBsAg and HBV DNA clearance has shown considerable variability across different studies (24). Regarding HBsAg clearance, our findings are consistent with those of a randomized controlled trial that demonstrated no additional HBsAg decline when switching to TDF in patients previously treated with ETV (25). In contrast, another open-label, active-controlled study reported a significantly higher rate of HBsAg loss at week 72 in patients treated with TDF plus PEG-IFNα for 48 weeks compared to those receiving monotherapy with either TDF or PEG-IFNα (26). This variability suggests the complex dynamics of HBsAg clearance and the influence of combination therapies. As for HBV DNA suppression, it has been well established that adding NAs such as TDF or ETV to PEG-IFNα regimens enhances the overall antiviral response, particularly in reducing HBV DNA levels (27). A study involving 130 HBV patients found that, in both entire and propensity score-matched cohorts, TDF therapy was associated with greater liver function protection and more pronounced HBV DNA load reduction compared with ETV therapy, though no differences were observed in recurrence rates or overall survival (28). However, a systematic review comparing the efficacy of TDF and ETV on HBV DNA suppression found no significant difference in their ability to achieve undetectable HBV DNA levels (29). The results from this current study indicate that while both TDF and ETV are effective in suppressing HBV replication, the nuances of their efficacy, particularly in combination therapies, warrant further investigation to optimize treatment outcomes for patients with chronic HBV infection.

### Results for propensity score-matched analysis

Our propensity score-matched analysis revealed statistically significant differences in HBV DNA clearance between the TDF combination therapy group and the IFN-α monotherapy group, suggesting superior antiviral efficacy with combination therapies. Despite IFN-α being approved as a first-line treatment for CHB in many countries, its use as a standalone antiviral therapy presents certain limitations. Studies have shown that among treatment-naïve CHB patients receiving PEG-IFNα-2b for over 1 year, only 10%–30% achieve HBeAg seroconversion and HBsAg clearance (30). Furthermore, during the course of PEG-IFNα-2b antiviral therapy, fluctuations in liver function parameters are common due to its stimulatory effects on the immune system (31). According to the results of this study, combining NAs with PEG-IFNα-2b to enhance its efficacy in improving hepatic histopathology has been further validated as a more reliable approach for the treatment of CHB.

Interestingly, the cumulative probability of HBsAg clearance remained statistically nonsignificant across the three treatment groups, further confirming the difficulty of achieving HBsAg loss even with potent antiviral agents. Nonetheless, when HBsAg levels were analyzed as a continuous variable, both the ETV and TDF combination groups demonstrated significantly more significant reductions in HBsAg levels than the IFN-α monotherapy group (*P* < 0.05). It is well documented that PEG-IFN combined with NAs can result in a functional cure for patients and improve the negative conversion rate of HBsAg (32, 33). On the other hand, clinical trials have demonstrated that the addition of PEG-IFN to NAs decreased HBsAg levels further and faster compared to NA monotherapy (14, 34). This finding underscores the utility of combination therapies in reducing HBsAg, despite the limited success in achieving full clearance.

Our analysis also indicated that the rate of ALT normalization was significantly higher in the ETV combination therapy group compared to the IFN monotherapy group (76.92% vs 45.45%, *P* < 0.05), suggesting a more favorable profile for liver enzyme recovery with ETV-based regimens. NAs, especially new generation NAs including ETV, have been illustrated to significantly inhibit viral replication to undetectable levels in more than 80% of treated patients and normalize ALT in almost all following long-term therapy (35). The discontinuation of NAs in CHB has been identified to induce HBV re-replication, often considered an undesirable virological relapse, and ALT flares (36).

Finally, the ETV combination therapy group had a significantly lower HBeAg clearance rate than IFN-α monotherapy (30.00% vs 87.50%, *P* < 0.05) in this study. Although ETV has well-known antiviral efficacy, the HBeAg seroconversion facilitated by ETV therapy remains relatively low. For instance, it was found that only about 20% of HBeAg-positive patients achieved HBeAg seroconversion after 48 weeks of ETV therapy (37). Comparative efficacy research showed that the addition of PEG-IFN to ETV in HBeAg-positive virally suppressed patients resulted in significantly more significant declines in HBeAg levels than in patients remaining on ETV monotherapy (38). Further studies are needed to explore the mechanisms behind this result. In contrast, no statistically significant differences in the efficacy of TDF combination therapy relative to the other treatment groups were observed in this current study, indicating a comparable clinical profile regarding overall treatment response for TDF.

### Adverse events

Adverse events were reported across all treatment groups, although incidence and persistence varied. Notably, the TDF combination therapy group exhibited a relatively higher frequency of adverse events, such as elevated ALT and AST levels and thrombocytopenia, compared to the ETV combination and IFN monotherapy groups. This finding is consistent with previous reports that have linked TDF to renal toxicity and osteomalacia (21). A previous study comprehensively analyzed the antiviral efficacy, adverse reactions, and emergence of resistance following 48 weeks of TDF treatment. This study found that TDF was generally well tolerated, with only a few patients discontinuing treatment due to adverse events, and no life-threatening adverse events were reported. The majority of recorded adverse reactions were related to gastrointestinal and renal issues (20).

While the TDF combination group showed an increased incidence of liver enzyme abnormalities, its efficacy in achieving viral suppression remains robust. Considering these facts, attempting to implement the appropriate treatment regimen may enhance the efficacy of TDF while reducing adverse effects. A study prospectively recruited 20 pregnant women with hepatitis B, dividing them into two subgroups: one starting TDF treatment in the second trimester and the other starting in the third trimester. The results indicated that initiating TDF treatment in the second trimester was more effective in suppressing the virus without increasing additional adverse reactions than starting treatment in the third trimester (39). Besides, TAF can be utilized as an alternative therapeutic strategy, which aims to maintain the efficacy of TDF while mitigating its negative impact on bone and kidney health (40).

In contrast, the ETV combination group demonstrated a more favorable safety profile regarding liver function recovery, suggesting that ETV may be a preferable option for patients at risk of liver enzyme elevations. The safety profile of TDF, particularly concerning its association with hepatic and renal toxicity and bone mineral density loss during long-term use, features the necessity of individualizing treatment regimens to balance efficacy with potential adverse events, especially for patients at higher risk of treatment-related toxicities.

This study comprehensively investigated the efficacy and safety profiles of IFN monotherapy, ETV combining IFN therapy, and TDF combining IFN therapy in patients with CHB. However, several limitations exist. First, as this is a real-world study, clinicians may have opted for different treatment strategies based on the specific conditions of individual patients. As a result, baseline HBV DNA and HBsAg levels were not comparable across different treatment groups in the entire analysis population. Although propensity score matching was used to reduce selection bias, unmeasured confounding factors may still have influenced the results. Second, the study included a limited number of subjects, leading to smaller group sizes after propensity score matching, which may have affected the outcomes. Future studies should be conducted in larger cohorts or through systematic reviews to validate these findings further. Third, while adequate for initial comparisons, the study’s duration may not be sufficient to capture the long-term benefits and risks of each treatment strategy.

### Conclusions

Overall, this study highlights the comparable efficacy of TDF and ETV combination therapies for viral suppression, with TDF showing superior HBV DNA clearance but a higher incidence of specific adverse events. Although there was no significant difference in HBsAg clearance among the treatment groups, both TDF and ETV demonstrated significant reductions in HBsAg levels compared to IFN monotherapy. Patients of TDF combination therapies with lower baseline HBsAg levels, high ALT levels, and lower APRI scores were more likely to achieve HBsAg loss. Further research with extended follow-up periods is warranted to better assess the full spectrum of efficacy and safety for these treatment regimens.

## Data Availability

The data used in this study can be accessed at https://github.com/hongxming/Spectrum02694. The results of this study are available on request from the corresponding authors, Dr Lin and Dr Chen.

## SUPPLEMENTAL MATERIAL

Supplemental tables spectrum.02694-24-s0001.docx Tables S1 and S2.

ASM does not own the copyrights to Supplemental Material that may be linked to, or accessed through, an article. The authors have granted ASM a non-exclusive, world-wide license to publish the Supplemental Material files. Please contact the corresponding author directly for reuse.
