# Supplementary material for: Comparative efficacy and safety of pegylated interferon-alpha monotherapy vs combination therapies with entecavir or tenofovir in chronic hepatitis B patients
Source: Microbiol Spectr. 2025 Apr 2;13(5):e02694-24. doi: 10.1128/spectrum.02694-24 (PMC12054097; doi:10.1128/spectrum.02694-24)
Supplement: Supplemental tables — Tables S1 and S2. [file spectrum.02694-24-s0001.docx]

**Supplementary Tables**

**Supplementary table 1. Baseline characteristics after trimatch**

|  | ETV+PEG-IFNα-2b | TDF+PEG-IFNα-2b | PEG-IFNα-2b | *P* |
| --- | --- | --- | --- | --- |
| N | 19 | 19 | 19 |  |
| Age,mean (SD), years | 37.05±10.37 | 34.95±7.00 | 40.95±8.98 | 0.118 |
| Sex |  |  |  |  |
| female | 6 (31.6) | 2 (10.5) | 5 (26.3) | 0.274 |
| male | 13 (68.4) | 17 (89.5) | 14 (73.7) |  |
| BMI,mean (SD),kg/m2 | 23.89±4.43 | 23.71±3.28 | 23.45±3.36 | 0.846 |
| Family history of HBV | 8 (42.1) | 3 (15.8) | 6 (31.6) | 0.203 |
| Family history of cancer | 6 (31.6) | 1 ( 5.3) | 7 (36.8) | 0.053 |
| Fatty liver | 10 (52.6) | 6 (31.6) | 10 (52.6) | 0.323 |
| HBV DNA, mean(SD), log10 IU/ml | 6.16±1.86 | 5.32±2.46 | 5.46±2.17 | 0.449 |
| HBsAg, mean(SD), log10 IU/ml | 3.70±0.79 | 3.67±0.77 | 3.54±0.91 | 0.818 |
| HBeAg status |  |  |  |  |
| negative | 9 (47.4) | 11 (57.9) | 11 (57.9) | 0.754 |
| positive | 10 (52.6) | 8 (42.1) | 8 (42.1) |  |
| ALT, mean (SD), U/L | 136.95±154.05 | 98.37±102.08 | 89.21±82.06 | 0.415 |
| AST, mean (SD), U/L | 77.37±70.72 | 50.89±42.09 | 60.47±58.63 | 0.374 |
| PLT, mean (SD), 10^9/L | 191.00±49.99 | 213.17±59.38 | 217.21±52.13 | 0.295 |
| FIB_4, mean (SD) | 1.42±0.76 | 0.99±0.77 | 1.31±1.00 | 0.301 |
| APRI, mean (SD) | 1.12±1.05 | 0.70±0.64 | 0.74±0.66 | 0.227 |

**Supplementary Table 2. Efficacy Results at Weeks 24 and 48 for all participants**

| Response | ETV+PEG-IFNα-2b(n=31) | TDF+PEG-IFNα-2b(n=59) | PEG-IFNα-2b(n=57) |
| --- | --- | --- | --- |
| **HBsAg loss, n/N (*%*)** |  |  |  |
| Week 24 | 0/31 | 9/59 (15.25) | 6/57 (10.53) |
| Week 48 | 1/31 (3.23) | 13/59 (22.03) | 10/57 (17.54) |
| **HBsAg seroconversion, n/N (%)** |  |  |  |
| Week 24 | 0/31 | 7/59 (11.86) | 5/57 (8.77) |
| Week 48 | 0/31 | 12/59 (20.34) | 9/57 (15.79) |
| **HBsAg change from baseline, log10 IU/mL** |  |  |  |
| Week 24 | -2.30 | -1.62 | -1.25 |
| Week 48 | -2.11 | -1.73 | -1.33 |
| **HBV DNA <20 IU/ml, n/N (%)** |  |  |  |
| Week 24 | 17/31 (54.84) | 43/59 (72.88) | 34/57 (59.65) |
| Week 48 | 21/31 (67.74) | 49/59 (83.05) | 41/57 (71.93) |
| **Functional care, n/N (*%*)** |  |  |  |
| Week 24 | 0/31 | 9/59 (15.25) | 5/57 (8.77) |
| Week 48 | 1/31 (3.23) | 13/59 (22.03) | 10/57 (17.54) |
| **HBeAg loss, n/N (*%*)** |  |  |  |
| Week 24 | 8/20 (40.00) | 14/29 (48.28) | 12/14 (85.71) |
| Week 48 | 7/20 (35.00) | 15/29 (51.72) | 11/14 (78.57) |
| **ALT normalization, n/N (*%*)** |  |  |  |
| Week 24 | 8/17 (47.06) | 19/38 (50.00) | 11/35 (31.43) |
| Week 48 | 15/17(88.24) | 26/38 (68.42) | 22/35 (62.86) |
| **AST normalization, n/N (*%*)** |  |  |  |
| Week 24 | 9/15 (60.00) | 18/34 (52.94) | 10/24 (41.67) |
| Week 48 | 11/15 (73.33) | 20/34 (58.82) | 14/24 (58.33) |
